# Physical and Chemical Properties of Gluten-Free Biscuits Incorporated With Purple Sweet Potato Flour

**DOI:** 10.1155/ijfo/9955286

**Published:** 2025-07-15

**Authors:** Chaiyasit Punfujinda, Paponpat Pattarathitiwat, Rath Chombhuphan, Sermsri Songnearm, Poomipong Tula, Korawit Sakkaekaew, Amornrat Anunvrapong, Sawai Boukaew, Krittin Chumkaew

**Affiliations:** ^1^Faculty of Home Economics Technology, Rajamangala University of Technology Thanyaburi, Pathum Thani, Thailand; ^2^Faculty of Culinary Arts, Dusit Thani College, Bangkok, Thailand; ^3^Faculty of Liberal Arts, Rajamangala University of Technology Krungthep, Bangkok, Thailand; ^4^Faculty of Agricultural Technology, Songkhla Rajabhat University, Songkhla, Thailand

**Keywords:** antioxidant, bakery, biscuits, gluten-free, purple sweet potato

## Abstract

Gluten-free products have gained increased attention due to the growing prevalence of gluten sensitivity and celiac disease. This study is aimed at developing gluten-free biscuits by replacing corn flour (CF) with purple sweet potato flour (PSPF) at levels of 0%–50% and at investigating the effects on physical properties, bioactive compounds, antioxidant activity, nutritional composition, and sensory evaluation. The results showed that increasing PSPF significantly enhanced (*p* < 0.05) the levels of bioactive compounds and antioxidant activity. The PSPF50 sample exhibited the highest total phenolic content (302.58 *μ*g GAE/g dw) and flavonoid content (974.86 *μ*g QE/g dw), compared to the control sample, which contained only 5.34 *μ*g GAE/g dw and 27.78 *μ*g QE/g dw. The antioxidant inhibition activities of 84.08% (DPPH) and 93.39% (ABTS) at 0.5 mg/mL represented 3.7-fold and 3.8-fold increases over the control. PSPF50 also showed higher protein content (2.20%) and dietary fiber (4.43%) than the control sample (0.32% and 0.52%, respectively). Significant changes were also observed in the spread ratio (8.82–12.02) and the density (0.86–1.54 g/cm^3^). Sensory evaluation indicated that the PSPF30 sample received the highest scores for all attributes, with ratings in the excellent range. Both lower and higher substitution levels led to a significant decline in acceptance (*p* < 0.05). In conclusion, PSPF enhances both the nutritional and functional properties of gluten-free biscuits. Substituting 30% PSPF was found to provide the best balance of sensory quality and industrial applicability.

## 1. Introduction

Gluten-free products have gained popularity due to consumer preferences related to health and trends [1]. They are particularly important for individuals with gluten intolerance or celiac disease, a lifelong gluten sensitivity that affects approximately 1% of the global population [2–5]. As the only treatment for this condition involves rigorous adherence to a gluten-free diet, there has been a surge in the demand for gluten-free food items [3, 6, 7].

Biscuits rank among the most extensively consumed products in the bakery industry [8]. In Thailand, biscuits are readily available in local convenience stores. Their popularity stems from their nutritional value, affordability, convenience, diverse flavors, ease of consumption, and long shelf life [3, 9–11]. The main ingredient in biscuits is wheat flour, which contains gluten proteins [12, 13]. Gluten is a key ingredient that plays a pivotal role in determining the characteristics of baked products, contributing to structure formation, adhesion, elasticity, dough strength, and product texture [12, 14, 15]. However, gluten structure formation is not essential in biscuit production, making it easier to replace wheat flour in biscuits with gluten-free alternatives compared to other gluten-dependent baked goods, like bread [2]. Consequently, gluten-free flours such as corn flour (CF) and purple sweet potato flour (PSPF) have emerged as promising ingredients for developing gluten-free biscuits.

CF is a gluten-free alternative [3] that is readily available and more affordable than wheat flour in Thailand. Recently, there has been a growing interest in developing gluten-free bakery products using CF, such as biscuits with varying ratios of corn, rice, and soybean flours, which affect nutritional value and sensory quality [3]. Additionally, biscuits made from rice, corn, and sorghum flours prepared with different ingredients have been studied for their physical properties [16]. Olaimat et al. [17] developed gluten-free corn-based biscuits supplemented with walnut and peanut.

Before that, PSPF had begun to gain popularity in gluten-free bakery production [18] as a partial replacement for main flour ingredients. Previous research has explored the development of biscuits using PSPF to replace wheat flour in different ratios [19], gluten-free biscuits from PSPF and other gluten-free flours in varying proportions [20], and cookies and muffins using PSPF, CF, and other flours as substitutes for wheat flour [21]. Furthermore, PSPF has garnered significant interest in terms of nutrition due to its rich phytochemical content, particularly its potent antioxidant and anti-inflammatory properties [18, 22–25]. It also contains dietary fiber, minerals, and anthocyanins, a subclass of flavonoids with high antioxidant capacity [26, 27].

Given the benefits of CF as a gluten-free alternative and the high nutritional value of gluten-free PSPF, it is essential to develop gluten-free biscuit products with enhanced functionality. In this development, the physical quality aspects and nutritional benefits of the biscuits should be thoroughly evaluated to ensure product quality and stability. These parameters are crucial indicators for product development, particularly for consumers with celiac disease who require safe and nutritious gluten-free alternatives. Consistent with the literature, the focus should be on developing functional gluten-free alternatives with improved physical and nutritional properties rather than replicating the exact properties of wheat-based products [28].

While previous studies have explored the use of CF and PSPF as replacements for wheat flour in biscuits and other bakery products, there has been a lack of research on the development of gluten-free biscuits made from PSPF as a substitute for CF, particularly in terms of physical quality, nutritional properties, and antioxidant capacity. This study is aimed at addressing this research gap by focusing on the development of gluten-free biscuit products using PSPF as a replacement for CF, with the goal of providing health benefits, offering an alternative product for gluten-sensitive consumers, and promoting new bakery products made from gluten-free flour.

## 2. Materials and Methods

### 2.1. Source of the Raw Materials

The CF (McGarrett), fresh purple sweet potato purchased from a local market in Pathum Thani province, sugar (Lin), butter (Allowrie), baking powder (Best Foods), salt (Prung Thip), and water (Crystal) were purchased from a supermarket in Pathum Thani province, Thailand.

### 2.2. Processing of Purple Sweet Potato to Flour

The purple sweet potatoes were washed, trimmed, and peeled to make them free from soil, rotting, or insect damage. They were cut into 1-in. thickness cubes before being steamed for 20 min. Sweet potato cubes were mashed, spread evenly on different trays, and then dried using a hot air dryer (Dryer machine O.V.D Series 15 Trays, Thailand) at 65°C for 24 h, following a modified method of Van Toan and Anh [19]. The dried samples were randomized and analyzed for moisture content (not exceeding 10%) using a moisture analyzer (METTLER TOLEDO, Model HE53, Switzerland) then milled into flour using Powder Grinder Model PG-ECO-1500 (32,000 r/min) and passed through a 250-*μ*m sieve to obtain uniform-sized flour. The flour was then packed in a sealed plastic bag and stored at ambient temperature until further used.

### 2.3. Preparation of Gluten-Free Biscuits

For this experiment, the researcher produced six samples of gluten-free biscuits. The variable factor was the ratio of PSPF to CF, as shown in Table 1: control: biscuits with 100% CF, PSPF10: biscuits with 10% PSPF replacing CF, PSPF20: biscuits with 20% PSPF replacing CF, PSPF30: biscuits with 30% PSPF replacing CF, PSPF40: biscuits with 40% PSPF replacing CF, and PSPF50: biscuits with 50% PSPF replacing CF. The preparation and production of the gluten-free biscuits were performed as follows. The biscuit preparation method was adapted from Kohli et al. [3] and Van Toan and Anh [19], with several adjustments made to suit the raw materials and equipment available. These included minor modifications to ingredient proportions, mixing technique, and baking conditions, and each gluten-free biscuit sample was prepared and produced using the same process. To ensure reproducibility, each sample was prepared in triplicate. The gluten-free biscuit production process was begun by creaming 100 g of butter using a mixer (KitchenAid, Model 5KPM5EER, United States) with a paddle attachment at medium speed until slightly fluffy. Then, 100 g of granulated sugar was added and mixed until a creamy consistency was achieved. CF and PSPF were combined according to the ratios shown in Table 1, along with 5 g of baking powder and 2 g of salt, and sifted together using a flour sifter. The sifted mixture was added to the creamed butter and sugar mixture, followed by 50 g of water, and the two mixtures were mixed together until they were well combined.

**Figure 1:**
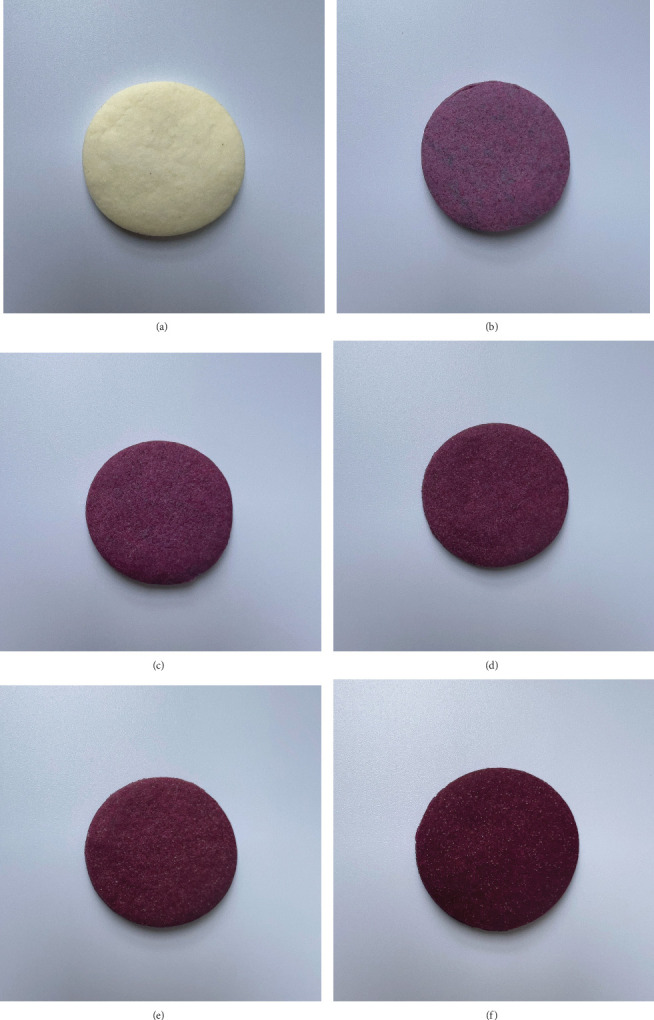
Gluten-free biscuit products made from purple sweet potato flour replacing corn flour in different ratios: (a) control, (b) PSPF10, (c) PSPF20, (d) PSPF30, (e) PSPF40, and (f) PSPF50.

The dough was kneaded until a smooth ball was formed, and then, it was refrigerated for 1 h. After chilling, the dough was evenly rolled onto a cutting board using a rolling pin. The gluten-free biscuits were cut into round shapes using a circular mold cutter (4 cm in diameter) and baked in a preheated laboratory baking oven (Piron, Model PF5004F, Italy) at 170°C for 20 min. The finished gluten-free biscuit products were obtained as shown in Figure 1. The gluten-free biscuits were then allowed to cool at room temperature for 1 h. Subsequently, the biscuits were packaged in labeled 200-*μ*m thick aluminum foil bags (250 g per bag) and heat-sealed. The packaged biscuits were kept at room temperature (25°C ± 2°C) during analysis.

### 2.4. Color Measurement

The color characteristics of raw materials (CF and PSPF) and gluten-free biscuits were analyzed using a HunterLab colorimeter (Colorflex EZ 45-0 [LAV], United States) with three replications. Each sample was prepared by placing 40 g into a glass cup for measurement (gluten-free biscuits were finely ground before measurement). The color parameters were recorded as *L*∗ (lightness; 0 = black, 100 = white), *a*∗ (red–green axis; positive = red, negative = green), and *b*∗ (yellow–blue axis; positive = yellow, negative = blue). The total color difference (Δ*E*) was calculated using a calibrated white tile as a reference standard [29].

 $$\begin{array}{c} \Delta E=\sqrt{{\left(L_{1}^{*}-L_{2}^{*}\right)}^{2}+{\left(a_{1}^{*}-a_{2}^{*}\right)}^{2}+{\left(b_{1}^{*}-b_{2}^{*}\right)}^{2}}. \end{array}$$

### 2.5. Texture Analysis

The hardness of gluten-free biscuits was determined using a texture analyzer (model LF Plus, LLOYD, England) with a 5 kg load cell and a P/2-2 mm diameter cylinder probe. Each biscuit sample (4 cm in diameter) was placed centrally on the platform. Prior to testing, all samples were equilibrated to room temperature (25°C ± 2°C) for 15 min to ensure uniform testing conditions. The instrument was calibrated for force using a standard 1 kg weight before measurement. The test conditions were set at a pretest speed of 1.0 mm/s, test speed of 0.5 mm/s, and posttest speed of 10.0 mm/s with 2 mm compression distance. The measurements were performed with three replications for each sample [30].

### 2.6. Baking Loss

The baking loss percentage of gluten-free biscuits was determined by weighing three sets of biscuits before and after baking. The initial weight (*W*0) was measured immediately after shaping the gluten-free biscuit dough. After baking and cooling to room temperature, the final weight (*W*1) was measured. The percentage (%) of baking loss was calculated, where *W*0 is the initial weight of the raw gluten-free biscuit dough and *W*1 is the final weight of the baked gluten-free biscuit, using the following equation [29].

 $$\begin{array}{c} \text{Banking loss }\left(\%\right)=\frac{W0-W1}{W0}\times 100. \end{array}$$

### 2.7. Diameter

The diameter of gluten-free biscuits was measured by placing six separate biscuits edge-to-edge horizontally. The measurements were taken with three replications using a digital vernier caliper and expressed in millimeters. The average diameter was calculated from these measurements [17].

### 2.8. Thickness

The thickness of gluten-free biscuits was determined by stacking six biscuits vertically. The measurements were taken with three replications using a digital vernier caliper and expressed in millimeters. The average thickness was calculated by rearranging and restacking the biscuits [17].

### 2.9. Spread Ratio

The spread ratio was calculated as the ratio of average diameter to average thickness (diameter/thickness) of gluten-free biscuits. The measurements were performed in triplicate for each sample [17, 20].

### 2.10. Weight

The weight of gluten-free biscuits was determined using a digital analytical balance with three replications. The average weight of six biscuits was measured and expressed in grams [17].

### 2.11. Volume and Density

The volume of gluten-free biscuits was defined as the cross-sectional area multiplied by thickness. The density was calculated as the ratio of weight to volume (AACC 1983) [19, 31].

 $$\begin{array}{c} \text{Volume }\left({\text{cm}}^{3}\right)=\frac{d^{2}\;\pi \;t}{4}, \end{array}$$

where *d* is the diameter of gluten-free biscuits (centimeter) and *t* is the thickness of gluten-free biscuits (centimeter).

 $$\begin{array}{c} \text{Density }\left(\frac{\text{g}}{{\text{m}}^{3}}\right)=\frac{\text{weight of sample }\left(\text{g}\right)}{\text{volume of sample }\left(\text{c}{\text{m}}^{3}\right)}. \end{array}$$

### 2.12. Total Phenolic Content (TPC) and Total Flavonoid Content (TFC)

Both raw materials (CF and PSPF) and gluten-free biscuits were analyzed with three replications. Sample extraction was performed over 2 days by using 200 mL of methanol to extract the powdered samples, each of which weighed approximately 100 g (2:1 ratio). Each extraction solution was spun in a centrifuge at 6000 rpm for 15 min. After that, the supernatant was filtered through a 0.45-*μ*m syringe filter before the levels of bioactive compounds could be determined [32].

To determine the TPC, 100 *μ*L of each sample solution was mixed with 100 *μ*L of methanol and 200 *μ*L of 10% (*v*/*v*) Folin–Ciocalteu reagent before it was shaken for 5 min. After that, 600 *μ*L of 1 M sodium carbonate was added to the solution mixture. The reaction mixture was incubated in darkness at room temperature for 60 min. A spectrophotometer was used to analyze the final product at 760 nm. The calibration curve of gallic acid (micrograms of gallic acid equivalent per gram of dry weight) was used to determine the TPC of each sample. To determine the TFC, 500 *μ*L of each sample solution was mixed with 340 *μ*L of deionized water and 30 *μ*L of sodium acetate (1 M) and incubated for 5 min. Then, the reaction solution was mixed with 30 *μ*L of AlCl_3_ (1 M) and shaken for 5 min. After that, 200 *μ*L of NaOH (1 M) was added, and the mixture was incubated for 15 min at 30°C. Lastly, a spectrophotometer was used to determine the absorbance of the final product at 415 nm. The calibration curve of quercetin (micrograms of quercetin equivalent per gram of dry weight) was used to measure the TFC of each sample solution [32].

### 2.13. Antioxidant Assay

Both raw materials (CF and PSPF) and gluten-free biscuits were analyzed with three replications. Sample extraction was performed over 2 days by using 200 mL of methanol to extract the powdered samples, each of which weighed approximately 100 g (2:1 ratio). Each extraction solution was spun in a centrifuge at 6000 rpm for 15 min. After that, the supernatant was filtered through a 0.45-*μ*m syringe filter. The solvent solutions were placed in a hot air oven heated to 50°C to evaporate off the liquid and leave behind the crude extracts. The crude extracts were stored at −20°C until they were required for further experiments [32].

Crude samples weighing approximately 1 g each were dissolved in 1 mL of DMSO (10% [*v*/*v*]) to produce solutions of 1 mg/mL in concentration. A 50 *μ*L portion of each sample solution was mixed with 50 *μ*L of DPPH solution (0.1 mM), resulting in samples of 0.5 mg/mL in terms of final concentration. Each reaction mixture was then stored in darkness at room temperature for 30 min. Radical inhibition absorbance was measured at 517 nm using a UV/Vis spectrophotometer. Meanwhile, the percentage of inhibition was determined by using [(*A*_blank_ − *A*_sample_)/*A*_blank_] × 100, where *A*_blank_ is the absorbance without sample and *A*_sample_ is the absorbance with sample. All samples underwent ABTS· + cation radical assay, for which the ABTS (7 mM) and potassium persulfate (2.45 mM) were mixed in a 1:0.5 *v*/*v* ratio to prepare the ABTS· + cation solution. Next, 50 *μ*L of each sample was mixed with 50 *μ*L of ABTS· + solution to a final concentration of 0.5 mg/mL in order to assess the inhibition potential of those samples against the ABTS· + cation radical. The resulting mixed solution was allowed to stand for 30 min at room temperature. Measurement was carried out at 734 nm to determine the inhibition potential. Meanwhile, a calculation similar to the DPPH assay calculation was performed to determine the inhibition percentage [32].

### 2.14. Proximate Composition Analysis

The proximate composition of raw materials (CF and PSPF) and gluten-free biscuits was analyzed with three replications according to AOAC [33] methods for moisture, ash, protein, fat, carbohydrate, and dietary fiber content.

### 2.15. Sensory Evaluation

Sensory evaluation of the gluten-free biscuit samples was conducted with 100 untrained panelists, all of whom were students from the Faculty of Culinary Arts, Dusit Thani College, Thailand. These panelists had prior exposure to gluten-free bakery products and had completed the pastry and bakery course as part of their curriculum. Written informed consent was obtained after participants were informed of the research objectives. All ingredients used were food grade and deemed safe for consumption. Participation was voluntary, and participants retained the right to withdraw at any time. No personal data were collected, and participant anonymity was strictly maintained [34–36]. Each panelist received one whole biscuit per sample. To maintain tasting accuracy and minimize sensory fatigue, drinking water was provided between samples. All biscuits were freshly prepared on the evaluation day. Sensory testing was performed in three replications to ensure result consistency. The study followed a randomized complete block design (RCBD). Panelists assessed six attributes: appearance, color, smell, taste, texture, and overall acceptability, using a 9-point hedonic scale ranging from 1 (*dislike extremely*) to 9 (*like extremely*). Intermediate descriptors such as *reasonable* (2–4), *good* (5–6), and *excellent* (7–8) were used to guide evaluations [3, 37].

### 2.16. Statistical Analysis

Data were expressed as mean ± standard deviation of three replications. Paired sample *t*-test was used to compare means between CF and PSPF samples. Analysis of variance (ANOVA) followed by Duncan's new multiple range test (DNMRT) was used to determine significant differences among gluten-free biscuit samples. Prior to conducting the paired *t*-test and ANOVA, the assumptions of normality and homogeneity of variances were checked using the Shapiro–Wilk test and Levene's test, respectively, to ensure the validity of the statistical analyses. Statistical significance was established at *p* < 0.05 using SPSS Software Version 22 (SPSS Inc., Chicago, Illinois, United States).

## 3. Results and Discussion

### 3.1. Physical Properties of Flour

The color values of CF and PSPF are shown in Table 2. The *L*∗ value, representing lightness, was significantly higher (*p* < 0.05) in CF (94.31) compared to PSPF (39.15), indicating that CF had a lighter color than PSPF. The *a*∗ value, representing redness, was significantly higher (*p* < 0.05) in PSPF (21.69) than in CF (−0.34), suggesting that PSPF had a more pronounced red hue. The *b*∗ value, representing yellowness, was significantly higher (*p* < 0.05) in CF (4.38) compared to PSPF (−9.15), indicating that CF had a more yellow color, while PSPF exhibited a blue hue. These differences in color values can be attributed to the presence of natural pigments, such as anthocyanins, in purple sweet potato [38–41]. These color characteristics may influence consumer perception. The darker, purplish appearance of PSPF may be associated with natural ingredients and higher nutritional value, particularly among health-conscious consumers. Such visual cues can enhance the product's appeal and increase its market potential in the functional and gluten-free food sectors [19, 42–44].

### 3.2. Bioactive Compounds, Antioxidant Activity, and Proximate Composition of Flour

The TPC and TFC were significantly higher (*p* < 0.05) in PSPF compared to CF (Table 2). The TPC of PSPF (567.51 *μ*g GAE/g dw) was approximately 124 times higher than that of CF (4.57 *μ*g GAE/g dw), while the TFC of PSPF (1195.65 *μ*g QE/g dw) was about 47 times higher than that of CF (25.21 *μ*g QE/g dw). These findings are consistent with previous studies reporting that purple sweet potato is rich in phenolic compounds and flavonoids, particularly anthocyanins [45–48]. The difference in antioxidant activity between PSPF and CF can be attributed to the higher amounts of phenolic compounds and flavonoids in PSPF. It is well known that these compounds possess strong antioxidant potential. This finding is consistent with previous studies showing that extracts from purple or dark red vegetables and fruits contain high levels of anthocyanins and phenolic compounds and exhibit excellent free radical scavenging abilities [49]. Furthermore, Fidrianny et al. [50] reported that extracts from purple sweet potatoes had higher total phenolic and flavonoid content compared to white sweet potatoes and exhibited significantly better antioxidant activities as measured by DPPH and FRAP assays. Therefore, it can be concluded that PSPF contains high amounts of phenolic compounds and flavonoids, which are the main factors contributing to its superior antioxidant properties compared to CF [50]. This makes PSPF a more promising ingredient for food applications, in terms of enhancing nutritional value and promoting health benefits, than CF. These benefits have been attributed to the presence of bioactive compounds in purple sweet potato, particularly flavonoids and phenolic acids, which have been reported to possess antioxidant, anti-inflammatory, immunomodulatory, antidiabetic, and antitumor properties [21, 43, 51–53]. Incorporating PSPF into gluten-free biscuits may therefore offer not only nutritional improvements but also added functional value that supports consumer health.

Regarding proximate composition, PSPF showed a significantly lower (*p* < 0.05) moisture (6.49%) compared to CF (11.90%). Additionally, PSPF contained a significantly lower (*p* < 0.05) carbohydrate content (77.22%) than CF (87.20%). However, PSPF exhibited significantly higher (*p* < 0.05) levels of ash (2.62%), protein (6.68%), and dietary fiber (6.92%) compared to CF (0.07%, 0.34%, and 0.16%, respectively). Conversely, the fat content of PSPF (0.07%) was significantly lower (*p* < 0.05) than that of CF (0.33%). These findings emphasized the superior nutritional profile of PSPF compared to CF, particularly in terms of its higher protein, ash, and dietary fiber contents, which could contribute to the overall nutritional quality and health benefits of food products. The increased dietary fiber content in PSPF might also play a role in promoting digestive health [54–56].

### 3.3. Physical Properties of the Gluten-Free Biscuits

The physical properties of gluten-free biscuits with different PSPF content are shown in Table 3. The color value results showed that the *L*∗ value, representing lightness, significantly decreased (*p* < 0.05) with increasing PSPF content in the gluten-free biscuits. The control sample (100% CF) had the highest *L*∗ value (85.55), while PSPF50 (50% PSPF) had the lowest (28.26). The *b*∗ value, representing yellowness, significantly decreased (*p* < 0.05) with increasing PSPF content in the gluten-free biscuits. The control sample had the highest *b*∗ value (22.10), indicating a more yellow color, while PSPF50 had the lowest (−3.28). Meanwhile, the *a*∗ value, representing redness, significantly increased (*p* < 0.05) with increasing PSPF content in the gluten-free biscuits. The control sample had the lowest *a*∗ value (2.51), while PSPF50 had the highest (26.60). These changes may be attributed to the anthocyanins present in PSPF [57, 58]. The deep purple hue of the sweet potatoes used in this study was attributed to the high concentration of acylated anthocyanins, particularly those derived from peonidin and cyanidin [59]. The results of this experiment were consistent with previous studies on color value testing of products containing PSPF [58, 60]. The progressive increase in total color difference (Δ*E*) with increasing PSPF content was significant (*p* < 0.05), demonstrating the substantial impact of PSPF incorporation on product appearance. These color changes remained stable after baking, which according to Jiang et al. [57] is due to the thermal stability of purple sweet potato anthocyanins compared to other natural pigments. Total color difference values increased significantly (*p* < 0.05) from 42.17 in PSPF10 to 67.11 in PSPF50. This characteristic makes PSPF particularly suitable for baked products where color stability during thermal processing is desired. The findings are consistent with Zhu and Sun [58] who reported similar color stability in PSPF-fortified steamed bread products after heat treatment. These noticeable changes in biscuit coloration, particularly the emergence of a distinct purple hue at higher PSPF levels, may positively influence consumer perception of product uniqueness and natural appeal. The visual characteristics imparted by PSPF could potentially enhance consumer color acceptance, especially in markets that favor vibrant, naturally derived pigments in health-oriented food products [19].

The hardness of the gluten-free biscuits significantly increased (*p* < 0.05) with increasing PSPF content. The control sample had the lowest hardness value (776.09 gf), while PSPF50 had the highest (2575.26 gf). This increase in hardness may be due to the higher dietary fiber content of PSPF compared to CF, revealed by the dietary fiber content values shown in Table 2. Furthermore, the research findings are in line with a study by Zhu and Sun [58], which found that steamed bread with higher levels of PSPF supplementation resulted in a significant increase in hardness values. These findings demonstrate that replacing CF with PSPF in gluten-free biscuits significantly affects their physical properties. These textural changes, particularly the significant increase in hardness, could potentially affect consumer perception and acceptance.

The physical characteristics of gluten-free biscuits were significantly affected (*p* < 0.05) by PSPF incorporation. The baking loss significantly decreased with increasing PSPF content from 7.37% (control) to 6.06% (PSPF50) due to higher water binding capacity of PSPF (2.76 g/g) [19] compared to CF (2.11 g/g) [61]. This finding aligns with Tanyitiku et al. [29] who found that fiber-rich ingredients improved moisture retention during baking. The diameter significantly decreased from 4.32 (control) to 4.09 cm (PSPF50), and thickness significantly decreased from 0.49 (control) to 0.34 cm (PSPF50) with PSPF incorporation. Previous research by Tanyitiku et al. [29] reported similar diameter reduction in gluten-free biscuits when fiber-rich ingredients were incorporated. This reduction can be attributed to the higher water absorption capacity of PSPF, which affects dough viscosity and spreading characteristics during baking. Similarly, Van Toan and Anh [19] reported that higher levels of sweet potato flour substitution led to decreased biscuit thickness due to the weakening of the structural matrix, while spread ratio increased from 8.82 (control) to 12.02 (PSPF50). These changes in physical dimensions can be attributed to PSPF's higher fiber content and water absorption capacity affecting dough behavior during baking, as reported by Van Toan and Anh [19] who found that spread ratio tends to increase significantly with increasing sweet potato flour substitution due to interference with gluten network formation. The spread ratio is commonly used to assess the rising ability of biscuits, with a lower ratio indicating a better rising capacity [62, 63]. These findings suggest that samples with a higher spread ratio tended to exhibit increased hardness, which may negatively influence consumer acceptability due to changes in texture and mouthfeel [17]. Weight increased from 6.23 (control) to 6.89 g (PSPF50) due to enhanced water retention capacity of PSPF's higher fiber content, consistent with Olaimat et al. [17] findings in gluten-free formulations. Volume showed a linear decrease while density increased with PSPF addition, with volume decreasing from 7.17 (control) to 4.46 cm^3^ (PSPF50). Van Toan and Anh [19] reported that this reduction in volume was possibly due to the fibers present in sweet potato flour interfering with matrix structure and diminishing gas retention capacity in the dough, leading to an increase in density from 0.86 (control) to 1.54 g/cm^3^ (PSPF50). Moreover, the decrease in volume and increase in density were similar to previous findings by Srivastava et al. [31] in their study of biscuits with wheat flour substituted by sweet potato flour.

### 3.4. TPC and TFC of the Gluten-Free Biscuits

The TPC and TFC of the gluten-free biscuits with different levels of PSPF are presented in Table 4. The TPC and TFC of the gluten-free biscuits significantly increased (*p* < 0.05) with increasing PSPF content. The control sample (100% CF) had the lowest TPC (5.34 *μ*g GAE/g dw) and TFC (27.78 *μ*g QE/g dw), while the PSPF50 sample (50% PSPF) had the highest TPC (302.58 *μ*g GAE/g dw) and TFC (974.86 *μ*g QE/g dw). These findings are consistent with previous studies reporting that purple sweet potato is a rich source of phenolic and flavonoid components [19, 64]. They are also consistent with the results of Bakar et al. [60], who found that the TPC and TFC of gluten-free biscuit products increased with the incorporation of higher proportions of purple sweet potato peel powder. Similarly, Zhu and Sun [58] reported that Chinese steamed bread products exhibited an increase in TPC when the proportion of PSPF was increased. The control sample had a TPC of 220 *μ*g GAE/g dw, while PSPF5%–PSPF100% samples had TPC values ranging from 510 to 6300 *μ*g GAE/g dw. Therefore, the variations in TPC and TFC among different samples can be attributed to the varying proportions of PSPF [3, 65]. These results suggest that incorporating PSPF into gluten-free biscuit formulations can be an effective strategy to increase the content of beneficial phenolic compounds and flavonoids in the final product.

### 3.5. Antioxidant Activity of the Gluten-Free Biscuits

The antioxidant activity of the gluten-free biscuits with different levels of PSPF, as measured by DPPH and ABTS assays, is presented in Table 4. The antioxidant activity of the gluten-free biscuits significantly increased (*p* < 0.05) with increasing PSPF content. The control sample had the lowest DPPH (22.77%) and ABTS (24.69%) inhibition percentages, while the PSPF50 sample had the highest DPPH (84.08%) and ABTS (93.39%) inhibition percentages at a concentration of 0.5 mg/mL. These results are consistent with previous studies reporting the high antioxidant capacity of PSPF [58, 60, 64, 66]. The incorporation of PSPF in gluten-free biscuits not only improves their nutritional value but also enhances their potential health benefits, such as reducing oxidative stress and preventing chronic diseases [59, 66, 67]. These findings highlight the potential of using PSPF as a functional ingredient in gluten-free biscuits to improve their antioxidant properties and overall health benefits.

In terms of anthocyanin stability, PSPF plays a significant role in enhancing the antioxidant activity of gluten-free biscuits, primarily due to its rich anthocyanin content. Although anthocyanins are widely recognized as natural, water-soluble pigments with potential applications in the food industry, their chemical structure makes them vulnerable to degradation. Environmental factors such as temperature, pH, light, metal ions, and redox agents can compromise their stability, leading to reduced antioxidant functionality and color loss [68, 69]. Compared with anthocyanins from other sources such as blackberry or grape skin, those found in purple sweet potato contain acylated and methylated forms that provide enhanced coloring ability and improved resistance to external stresses, making them more suitable for use in beverages, baked goods, and frozen products [24]. The improved stability of these compounds is attributed to phenolic acid acylation, which enhances tolerance to heat, pH variation, and light exposure [70]. Xu et al. [70] identified 12 acylated anthocyanins in the purple sweet potato variety P40 and found that cooking processes including steaming, pressure cooking, microwaving, and frying resulted in only 8%–16% loss of total anthocyanins, whereas baking showed little to no significant effect. This observation is supported by additional studies that highlight how the structural complexity of purple sweet potato anthocyanins contributes to their notable stability under heat and ultraviolet radiation [71, 72]. These characteristics indicate that PSPF represents a promising candidate for thermally processed functional foods, as it retains considerable pigment and antioxidant capacity after baking, although further investigation is warranted.

### 3.6. Proximate Composition of the Gluten-Free Biscuits

Table 5 shows the proximate composition of the gluten-free biscuits according to the level of PSPF. As increasing amounts of PSPF were added, the moisture of the gluten-free biscuit samples rose significantly (*p* < 0.05), varying from 1.83% in the control sample to 3.19% in the PSPF30 sample. As expected, the moisture of all tested samples was under 10% [3, 73]. Additionally, as increasing amounts of PSPF were added, the ash content also rose significantly (*p* < 0.05); the PSPF50 sample exhibited the highest ash (1.72%), while the lowest (0.89%) was observed in the control sample. This result was in line with a previous study on the use of different proportions of PSPF to replace wheat flour in biscuits [19]. Furthermore, the protein content also rose significantly (*p* < 0.05); the PSPF50 sample had the highest level (2.20%), while the lowest (0.32%) was found in the control sample. This result was consistent with another previous study on biscuits made with PSPF [60]. There was no significant difference in fat content (*p* ≥ 0.05), ranging from 18.20% to 18.70%. The carbohydrate content decreased significantly (*p* < 0.05) as PSPF amounts increased; the highest value (77.74%) was observed in the control sample and the lowest (70.15%) in PSPF50. The dietary fiber content increased significantly (*p* < 0.05); the highest value (4.43%) was observed in PSPF50 and the lowest (0.52%) in the control sample. This result confirmed those of previous research on biscuit and cracker products with PSPF added [19, 60, 74]. These findings showed that adding PSPF to gluten-free biscuits enhanced their nutritional profile, especially regarding ash, protein, and dietary fiber, while lowering carbohydrate levels. Consequently, the general nutritional value and possible health benefits of gluten-free biscuits were improved, suggesting that PSPF could become more widely used in the development of healthy foods [64].

### 3.7. Sensory Evaluation of the Gluten-Free Biscuits

**Figure 2:**
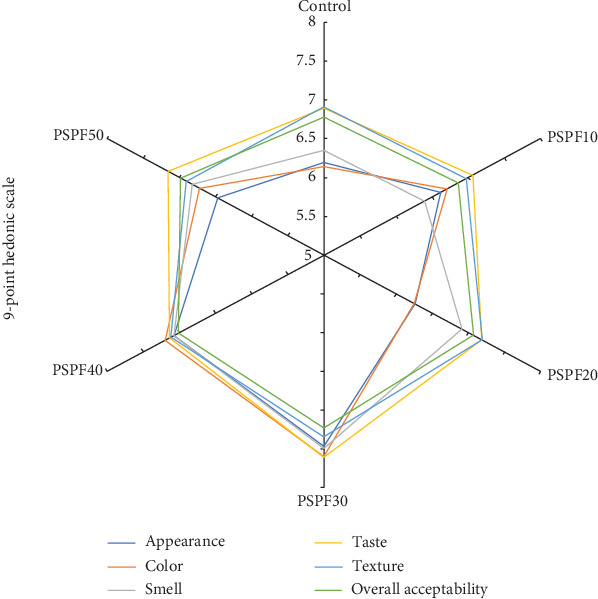
Sensory evaluation of different gluten-free biscuit samples.

Sensory evaluation results, as shown in Figure 2, revealed statistically significant differences among samples (*p* < 0.05). The PSPF30 achieved the highest mean scores across all attributes: appearance (7.47 ± 1.32), color (7.60 ± 1.40), smell (7.50 ± 0.96), taste (7.61 ± 1.44), texture (7.35 ± 1.48), and overall acceptability (7.23 ± 1.30), which were all rated as excellent. Overall acceptability scores showed that the control (6.78 ± 1.24), PSPF10 (6.86 ± 1.11), and PSPF50 (6.99 ± 1.48) were rated as good, while PSPF40 (7.02 ± 1.27), PSPF20 (7.07 ± 1.27), and PSPF30 (7.23 ± 1.30) achieved excellent ratings. Notably, both lower and higher substitution levels significantly reduced sensory preference (*p* < 0.05), suggesting that an optimal substitution range exists. These findings establish PSPF30 as the optimal formulation, delivering superior sensory performance with strong potential for industrial application. The results align with previous studies where partial PSPF substitution enhanced consumer preference [19], attributed to its natural pigmentation improving visual appeal [75]. However, excessive substitution may compromise texture and flavor due to high fiber content and moisture-retention properties [58]. Therefore, balanced formulation remains crucial for optimizing both nutritional benefits and sensory quality [48, 58].

The successful sensory acceptance of the PSPF30 formulation demonstrates strong potential for industrial-scale production. The processing conditions employed (170°C for 20 min) utilize standard bakery equipment and require no process modifications, facilitating seamless integration into existing production lines. The widespread cultivation of purple sweet potato in Thailand ensures a stable raw material supply, while the flour preparation method (steaming, drying at 65°C, and milling) comprises scalable unit operations. The PSPF30 sample's physical properties support industrial feasibility, exhibiting acceptable hardness levels (1342.37 gf) and reduced baking loss (6.51% compared to 7.37%), which could enhance production yield. Furthermore, the improved nutritional profile, particularly the 4.7-fold increase in dietary fiber and significant antioxidant activity, justifies premium positioning in the functional food market. However, pilot-scale trials and storage stability assessments remain essential for successful commercialization.

## 4. Conclusions

PSPF contains significant bioactive compounds and antioxidant activity, with lower carbohydrate content than CF. Its substitution in gluten-free biscuit production proved nutritionally advantageous, significantly influencing functional and physical properties. Blending PSPF with CF improved nutritional value, particularly protein and dietary fiber content, while maintaining acceptable physical characteristics. The 30% PSPF substitution achieved the highest overall acceptability scores, indicating optimal balance between nutritional enhancement and consumer preference. However, limitations include potential quality variations due to seasonal factors, growing conditions, and cultivar differences affecting product consistency. Scale-up production challenges and long-term storage stability concerns regarding anthocyanin degradation require consideration. While PSPF-based biscuits offer improved nutritional profiles with higher protein, ash, and fiber contents, these changes may influence taste and texture. These findings provide valuable insights for industries developing nutritionally enhanced gluten-free products using PSPF as an alternative to CF.

## Figures and Tables

**Table 1:** The gluten-free biscuit ingredients in different samples, measured in grams.

**Sample**	**Corn flour**	**Purple sweet potato flour**	**Butter**	**Sugar**	**Baking powder**	**Salt**	**Water**
100% corn flour (control)	250	—	100	100	5	2	50
Biscuit formulations with PSPF replacement							
10% PSPF (PSPF10)	225	25	100	100	5	2	50
20% PSPF (PSPF20)	200	50	100	100	5	2	50
30% PSPF (PSPF30)	175	75	100	100	5	2	50
40% PSPF (PSPF40)	150	100	100	100	5	2	50
50% PSPF (PSPF50)	125	125	100	100	5	2	50

Abbreviations: control, biscuits containing 100% corn flour; PSPF, purple sweet potato flour; PSPF10, PSPF20, PSPF30, PSPF40, and PSPF50, biscuits prepared by replacing 10%, 20%, 30%, 40%, and 50% of the corn flour with purple sweet potato flour, respectively.

**Table 2:** Color values, levels of bioactive compounds, antioxidant activity, and proximate composition of corn flour and purple sweet potato flour.

**Analysis**	**CF**	**PSPF**
*L*∗	94.31^a^ ± 0.03	39.15^b^ ± 0.04
*a*∗	−0.34^b^ ± 0.01	21.69^a^ ± 0.02
*b*∗	4.38^a^ ± 0.01	−9.15^b^ ± 0.01
TPC (*μ*g GAE/g dw)	4.57^b^ ± 0.18	567.51^a^ ± 3.35
TFC (*μ*g QE/g dw)	25.21^b^ ± 0.43	1195.65^a^ ± 4.67
DPPH (% inhibition at 0.5 mg/mL)	19.34^b^ ± 0.42	104.56^a^ ± 1.12
ABTS (% inhibition at 0.5 mg/mL)	20.51^b^ ± 1.49	112.55^a^ ± 0.67
Moisture (%)	11.90^a^ ± 0.10	6.49^b^ ± 0.29
Ash (%)	0.07^b^ ± 0.01	2.62^a^ ± 0.03
Protein (%)	0.34^b^ ± 0.01	6.68^a^ ± 0.07
Fat (%)	0.33^a^ ± 0.01	0.07^b^ ± 0.01
Carbohydrate (%)	87.20^a^ ± 0.45	77.22^b^ ± 0.27
Dietary fiber (%)	0.16^b^ ± 0.02	6.92^a^ ± 0.03

*Note:* Values are means ± standard deviations; each value in the table is the mean of three replications (*n* = 3). Values with different superscript letters within the same vertical column are significantly different at the 95% confidence level (*p* < 0.05).

Abbreviations: CF, corn flour; DPPH assay and ABTS assay, percentage of inhibition at a concentration of 0.5 mg/mL; PSPF, purple sweet potato flour; TFC, total flavonoid content (quercetin equivalent per gram of dry weight); TPC, total phenolic content (micrograms of gallic acid equivalent per gram of dry weight).

**Table 3:** Physical properties of gluten-free biscuits.

**Parameter**	**Control**	**PSPF10**	**PSPF20**	**PSPF30**	**PSPF40**	**PSPF50**
Color						
*L*∗	85.55^a^ ± 0.03	50.93^b^ ± 0.01	41.10^c^ ± 0.01	35.76^d^ ± 0.01	32.09^e^ ± 0.01	28.26^f^ ± 0.01
*a*∗	2.51^f^ ± 0.01	17.66^e^ ± 0.01	23.01^d^ ± 0.02	24.93^c^ ± 0.01	26.03^b^ ± 0.02	26.60^a^ ± 0.01
*b*∗	22.10^a^ ± 0.04	3.35^b^ ± 0.01	−1.62^c^ ± 0.01	−2.70^d^ ± 0.02	−2.98^e^ ± 0.02	−3.28^f^ ± 0.01
Δ*E*	—	42.17^e^ ± 0.03	54.38^d^ ± 0.02	59.96^c^ ± 0.03	63.55^b^ ± 0.02	67.11^a^ ± 0.01
Hardness (gf)	776.09^f^ ± 2.57	884.34^e^ ± 6.19	1137.91^d^ ± 6.11	1342.37^c^ ± 7.62	2055.49^b^ ± 7.84	2575.26^a^ ± 9.81
Baking loss (%)	7.37^a^ ± 0.05	7.20^b^ ± 0.02	6.69^c^ ± 0.04	6.51^d^ ± 0.02	6.38^e^ ± 0.02	6.06^f^ ± 0.02
Diameter (cm)	4.32^a^ ± 0.03	4.26^b^ ± 0.02	4.20^c^ ± 0.01	4.15^d^ ± 0.02	4.11^e^ ± 0.01	4.09^e^ ± 0.01
Thickness (cm)	0.49^a^ ± 0.02	0.43^b^ ± 0.02	0.40^c^ ± 0.01	0.38^c^ ± 0.01	0.35^d^ ± 0.01	0.34^d^ ± 0.02
Spread ratio (D/T)	8.82^d^ ± 0.30	9.91^c^ ± 0.41	10.50^bc^ ± 0.23	10.92^b^ ± 0.23	11.74^a^ ± 0.31	12.02^a^ ± 0.66
Weight (g)	6.23^f^ ± 0.03	6.42^e^ ± 0.03	6.58^d^ ± 0.04	6.69^c^ ± 0.04	6.80^b^ ± 0.03	6.89^a^ ± 0.02
Volume (cm^3^)	7.17^a^ ± 0.39	6.12^b^ ± 0.34	5.53^c^ ± 0.16	5.13^c^ ± 0.18	4.63^d^ ± 0.28	4.46^d^ ± 0.28
Density (g/cm^3^)	0.86^e^ ± 0.04	1.04^d^ ± 0.05	1.18^c^ ± 0.02	1.30^b^ ± 0.04	1.46^a^ ± 0.04	1.54^a^ ± 0.09

*Note:* Values are means ± standard deviations; each value in the table is the mean of three replications (*n* = 3). Values with different superscript letters within the same row are significantly different at the 95% confidence level (*p* < 0.05).

**Table 4:** Bioactive compounds and antioxidant activity in different samples of gluten-free biscuits.

**Sample**	**TPC (*μ*g GAE/g dw)**	**TFC (*μ*g QE/g dw)**	**DPPH (% inhibition at 0.5 mg/mL)**	**ABTS (% inhibition at 0.5 mg/mL)**
Control	5.34^f^ ± 0.53	27.78^f^ ± 0.40	22.77^f^ ± 0.19	24.69^f^ ± 0.36
PSPF10	128.04^e^ ± 0.57	591.26^e^ ± 3.69	28.44^e^ ± 0.18	55.94^e^ ± 0.43
PSPF20	175.96^d^ ± 3.74	726.35^d^ ± 5.63	44.61^d^ ± 0.50	60.88^d^ ± 0.38
PSPF30	241.60^c^ ± 5.12	819.47^c^ ± 2.99	58.29^c^ ± 0.48	68.88^c^ ± 0.65
PSPF40	279.68^b^ ± 4.73	895.42^b^ ± 2.82	75.95^b^ ± 0.83	79.80^b^ ± 0.85
PSPF50	302.58^a^ ± 2.13	974.86^a^ ± 4.21	84.08^a^ ± 1.06	93.39^a^ ± 0.64

*Note:* Values are means ± standard deviations; each value in the table is the mean of three replications (*n* = 3). Values with different superscript letters within the same vertical column are significantly different at the 95% confidence level (*p* < 0.05).

Abbreviations: DPPH assay and ABTS assay, percentage of inhibition at 0.5 mg/mL; TFC, total flavonoid content (quercetin equivalent per gram of dry weight); TPC, total phenolic content (micrograms of gallic acid equivalent per gram of dry weight).

**Table 5:** Proximate composition of different samples of gluten-free biscuits.

**Sample**	**Moisture (%)**	**Ash (%)**	**Protein (%)**	**Fat (%)** ^**ns**^	**Carbohydrate (%)**	**Dietary fiber (%)**
Control	1.83^b^ ± 0.07	0.89^d^ ± 0.07	0.32^e^ ± 0.03	18.70 ± 0.54	77.74^a^ ± 0.49	0.52^f^ ± 0.02
PSPF10	3.09^a^ ± 0.14	1.17^c^ ± 0.20	0.74^d^ ± 0.06	18.60 ± 1.16	75.43^b^ ± 0.28	0.97^e^ ± 0.03
PSPF20	3.12^a^ ± 0.14	1.49^b^ ± 0.10	1.30^c^ ± 0.11	18.30 ± 0.74	73.97^c^ ± 0.17	1.82^d^ ± 0.04
PSPF30	3.19^a^ ± 0.19	1.61^ab^ ± 0.06	1.51^bc^ ± 0.27	18.20 ± 0.25	73.04^d^ ± 0.19	2.45^c^ ± 0.05
PSPF40	3.04^a^ ± 0.15	1.67^ab^ ± 0.13	1.69^b^ ± 0.07	18.30 ± 0.15	71.56^e^ ± 0.36	3.74^b^ ± 0.04
PSPF50	3.10^a^ ± 0.14	1.72^a^ ± 0.07	2.20^a^ ± 0.16	18.40 ± 0.15	70.15^f^ ± 0.13	4.43^a^ ± 0.05

*Note:* Values are means ± standard deviations; each value in the table is the mean of three replications (*n* = 3). Values with different superscript letters within the same vertical column are significantly different at the 95% confidence level (*p* < 0.05). ^ns^ refers to a nonsignificant difference (*p* ≥ 0.05).

## Data Availability

The data that support the findings of this study are available from the corresponding author upon reasonable request.
